# The Gardener's Dictionary, &c.

**Published:** 1834-07-01

**Authors:** 


*The Gardener's Dictionary, &c.* By PHILIP MILLER, F.R.S. &c. A new Edition.—London, 1833 and 4. 8vo. Nos. 1—5.

ALAS, poor Philip Miller! Heaven save thee from thy friends! How much cause hast thou to bewail the indiscretions of thy editors. Martyn encumbered thee with ancient lore, and with too much learning made thy useful pleasant tome distasteful and unwieldy. Don has kept thy name as his foundation, but the superstructure is not thine,—thou wouldst not know it; and now two other adventurers have set sail in thy fair bark. As yet, they have scarcely launched out from the shore; what will be the fate of their adventures is in the womb of time; but, as the freight, in part, consists of unacknowledged transcripts, page after page, of the Saturday Magazine, we do not augur well of the rest of the cargo.

If the articles alluded to, viz. those on the Age of Trees, were worth embodying in the work, an acknowledgment should have been made, even when taken from a penny magazine; but column after column of letterpress, besides figures, are introduced, without even the commas of quotation.

This book is the chronological miracle already mentioned in our Journal: a number appears but once a month, yet fifty will be published within a year.

